# Three-material decomposition with dual-layer spectral CT compared to MRI for the detection of bone marrow edema in patients with acute vertebral fractures

**DOI:** 10.1007/s00256-018-2981-x

**Published:** 2018-05-25

**Authors:** Benedikt J. Schwaiger, Alexandra S. Gersing, Johannes Hammel, Kai Mei, Felix K. Kopp, Jan S. Kirschke, Ernst J. Rummeny, Klaus Wörtler, Thomas Baum, Peter B. Noël

**Affiliations:** 1 Department of Radiology, Klinikum rechts der Isar, Technical University of Munich, Ismaninger Str. 22, 81675 Munich, Germany; 2 Department of Neuroradiology, Klinikum rechts der Isar, Technical University of Munich, Munich, Germany; 3 Physics Department & Munich School of BioEngineering, Technical University of Munich, Munich, Germany

**Keywords:** Spine, Vertebral fractures, Osteoporosis, Dual-layer spectral computed tomography

## Abstract

**Objectives:**

To assess whether bone marrow edema in patients with acute vertebral fractures can be accurately diagnosed based on three-material decomposition with dual-layer spectral CT (DLCT).

**Materials and methods:**

Acute (*n* = 41) and chronic (*n* = 18) osteoporotic thoracolumbar vertebral fractures as diagnosed by MRI (hyperintense signal in STIR sequences) in 27 subjects (72 ± 11 years; 17 women) were assessed with DLCT. Spectral data were decomposed into hydroxyapatite, edema-equivalent, and fat-equivalent density maps using an in-house-developed algorithm. Two radiologists, blinded to clinical and MR findings, assessed DLCT and conventional CT independently, using a Likert scale (1 = no edema; 2 = likely no edema; 3 = likely edema; 4 = edema). For DLCT and conventional CT, accuracy, sensitivity, and specificity for identifying acute fractures (Likert scale, 3 and 4) were analyzed separately using MRI as standard of reference.

**Results:**

For the identification of acute fractures, conventional CT showed a sensitivity of 0.73–0.76 and specificity of 0.78–0.83, whereas the sensitivity (0.93–0.95) and specificity (0.89) of decomposed DLCT images were substantially higher. Accuracy increased from 0.76 for conventional CT to 0.92–0.93 using DLCT. Interreader agreement for fracture assessment was high in conventional CT (weighted κ [95% confidence interval]; 0.81 [0.70; 0.92]) and DLCT (0.96 [0.92; 1.00]).

**Conclusions:**

Material decomposition of DLCT data substantially improved accuracy for the diagnosis of acute vertebral fractures, with a high interreader agreement. This may spare patients additional examinations and facilitate the diagnosis of vertebral fractures.

## Introduction

Vertebral compression fractures are a common pathology found in patients with reduced bone mineral density (BMD) caused by osteoporosis [1], but also in otherwise healthy patients after adequate trauma. With a prevalence of up to 26% in women and 24% in men older than 49 years [1], vertebral fractures are associated with an age-adjusted 32% increased risk of mortality [2]. Adequate management of fractures not only depends on accurate morphologic assessment, including the evaluation of the stability of the affected spinal units, but also on accurate assessment of fracture age. Although several CT-based classification criteria for spine injuries exist [3], CT in clinical routine lacks accuracy regarding the determination of the age of vertebral fractures. Therefore, MR imaging is often performed to identify edema-equivalent bone marrow changes, which are considered highly specific for acute trauma [4–6]. However, MR imaging is associated with additional examination times and substantial costs, and some patients are not eligible due to contra-indications such as pacemakers, other implants, or severe pain.

Therefore, the detection of bone marrow edema with other modalities has received growing attention, with dual-energy CT (DECT) being one of the most promising techniques. DECT allows for exploiting spectral information and thus the estimation of object composition including reconstructions such as virtual non-calcium images [7]. There are different approaches to DECT such as fast kV-switching or using two X-ray sources with different characteristics, known as dual-source CT [8, 9]. These technologies, however, need dedicated examination protocols, may be affected by motion artifacts, and associated with increased radiation exposure [7].

Recently, a new technique with one X-ray tube and two different detector layers mounted upon each other and absorbing different energy spectra of the polychromatic X-ray spectrum, known as dual-layer spectral CT, was introduced [10–12]. This approach allows for routinely reconstructing spectral information without the use of a specific protocol, and applications for material decomposition such as the quantification of iodine as well as the reconstruction of iodine-free images (virtual non-contrast or VNC imaging) are already commercially available [13, 14].

Another application for material decomposition based on DECT is the reconstruction of virtual non-calcium (VNCa). Previous studies have demonstrated that VNCa images derived from dual-source DECT show significantly higher detection rates of traumatic bone marrow edema compared to conventional CT and thus provide additional information for the diagnosis of acute vertebral fractures [15–18].

The aim of this study was to apply a three-material decomposition algorithm generating attenuation maps specific for bone marrow edema to spectral imaging data of patients with vertebral fractures acquired on a dual-layer spectral CT in order to differentiate fractures with and without traumatic bone marrow edema and thus acute versus chronic fractures.

## Methods

### Patients

Institutional review board approval was obtained prior to this study (Ethikkommission der medizinischen Fakultät, Technical University of Munich, Munich, Germany). Written informed consent was waived for this retrospective analysis of routinely acquired imaging data.

In our institutional PACS, patients admitted between January and October 2017 for suspected acute vertebral fractures that had undergone a CT examination without i.v. contrast application on our dual-layer spectral CT scanner and an MR examination within 3 days after the CT scan, were identified retrospectively. Patients with either incomplete spectral CT datasets (*n* = 2) of with one of the following conditions were excluded from the analyses: presence of metal artifacts due to spinal augmentation (*n* = 2), a condition affecting bone or fat metabolism, such as malignant diseases or diseases requiring systemic glucocorticoid or hormone therapy other than osteopenia or osteoporosis (*n* = 1 with myeloma and* n* = 1 with metastatic breast cancer), and infectious diseases of the spine (*n* = 1 with clinical and imaging findings of an acute spondylodiscitis). A total of *n* = 27 patients was included in the analysis.

### CT imaging protocol and post-processing

Spectral CT images were acquired by using a dual-layer spectral CT scanner (IQon Spectral CT, Philips Healthcare, Best, The Netherlands).

A routine spine protocol with a fixed tube voltage of 120 kV was used for all examinations. Patient examinations were performed with different exposures from 37 to 300 mAs with an average exposure of 93 ± 73 mAs. The resulting CT Dose Indices (CTDIvol) recorded in the dose reports generated by the scanner ranged from 3.4 to 27.1 mGy with an average of 8.3 ± 6.4 mGy.

Spectral base image (SBI) data were reconstructed using both a standard filter (B) and a standard bone filter (YB) with an axial slice thickness of 0.9 mm. The field of view was 250 × 250 mm. To homogenize images and reduce noise, sagittal reformations were averaged up to a thickness of 3.0 mm.

SBI datasets contain information on energy-dependent absorption, extracted with dual-layer detector technology. This information can be used to create virtual monochromatic (MonoE) images at 50 and 200 keV showing HU information like from a monoenergetic X-ray source [19]. These images were generated with IntelliSpace Portal 9.0.1 (Philips Healthcare).

For further image calculations, an advanced post-processing platform (IntelliSpace Discovery 2.0, Philips Healthcare) was used. An in-house-developed plug-in was implemented to generate red marrow, yellow marrow, and hydroxyapatite volume fraction maps by applying a three-material decomposition on the spectral information from the MonoE images (plug-in available from the authors on request). Interest lies on the red marrow map, as it contains more than 90% of water and therefore looks similar to the MR STIR sequence map.

To decompose two linear-independent MonoE images into three-material volume fractions, the algorithm assumes volume conservation with these materials. It is assumed that the volume of a material mixture at a given temperature and pressure equals the sum of the volume of its constituent parts at the same temperature and pressure [20]. Representing this mathematically, the sum of the volume fractions needs to equal one. This assumption is used for image analysis algorithms like virtual non-enhanced images for CT urography and angiography, or liver-fat quantification.

Hounsfield units, the entity in MonoE images, are converted to attenuation coefficients using the formula

1 $$ \mu (E)=\frac{HU(E)\ast {\mu}_{water}(E)}{1000\ \left[ HU\right]}+{\mu}_{water}(E) $$

with *μ*_water_(*E*) the attenuation coefficient of water at energy *E* obtained from the United States National Institute of Technology and Standards (NIST) X-Ray Mass Attenuation Coefficients Database [21] and *HU*(*E*) the Hounsfield units at energy *E* given in the MonoE. The linear equation system for the image-based material decomposition can be written then as

2 $$ \mu \left({E}_1\right)={f}_1{\mu}_{m,1}\left({E}_1\right)+{f}_2{\mu}_{m,2}\left({E}_1\right)+{f}_3{\mu}_{m,3}\left({E}_1\right) $$

3 $$ \mu \left({E}_2\right)={f}_1{\mu}_{m,1}\left({E}_2\right)+{f}_2{\mu}_{m,2}\left({E}_2\right)+{f}_3{\mu}_{m,3}\left({E}_2\right) $$

4 $$ {f}_1+{f}_2+{f}_3=1 $$

where *μ*(*E*) is the measured attenuation coefficient at energy *E*_1_ (50 keV) or *E*_2_ (200 keV), *f*_1_, *f*_2_ and *f*_3_ are the volume fractions of red marrow, yellow marrow, and hydroxyapatite, and *μ*_1, 2, 3_(*E*) are the known attenuation coefficients of the corresponding materials in pure form. These mass attenuation coefficients are taken from the International Commission on Radiation Units and Measurements Report 46 [22]. By solving the linear equation system on a pixel-by-pixel base for *f*_1_, *f*_2_, and *f*_3_, 3D images for all material volume fractions are generated and can be analyzed in different anatomical planes. The computational process for one abdomen scan on the IntelliSpace Discovery Server for this takes about 30 s.

**Fig. 1:**
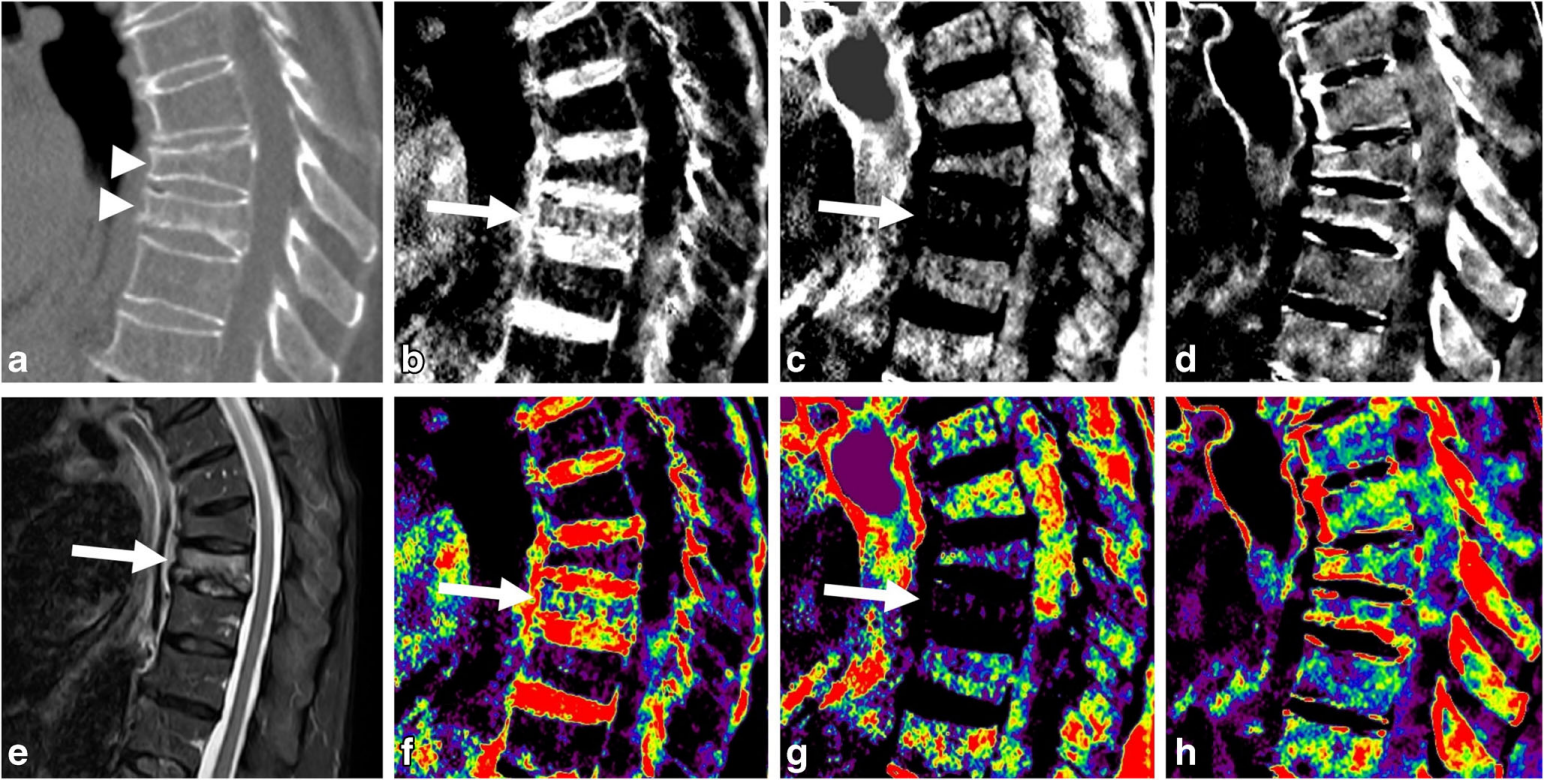
CT and MR images of a 67-year-old female patient admitted for acute back pain and history of osteopenia. Sagittal reformation equivalent to a conventional CT examination (**a**) reveals compression fractures of Th10 and 11 (*arrowheads*). Th11 presents a global increase in HU numbers possibly due to condensation, but no definite signs of an acute fracture are seen. Material-specific density maps for water (**b**), fat (**c**), and hydroxyapatite (**d**), and respective color-coded overlays (**f**,** g**,** h**) reveal an increase in water-specific density in the red-marrow map (**b** and** f**;* arrow*) and a decrease in fat-specific density in the yellow-marrow map (**c** and** g**;* arrow*) in Th11 compared to adjacent vertebral bodies, including Th10. The hydroxyapatite-specific map suggests a density increase in the lower endplate in Th11 (**d** and** h**). The sagittal fluid-sensitive fat-saturated STIR sequence from the subsequent MR examination confirms the presence of an edema-equivalent signal alteration in Th11, thus confirming an acute fracture (*arrow*), but not in Th10, thus confirming a chronic fracture (**e**)

Density maps for red marrow, yellow marrow, and hydroxyapatite volume fractions were generated in a sagittal orientation and a section thickness of 3 mm (Fig. 1). In addition to grayscale reformations, color-coded variants of density maps were calculated with a standard rainbow filter within the IntelliSpace Discovery software suite transferring whiter grayscale values to warmer colors (Fig. 1). As part of the standard-of-care protocol, conventional polychromatic CT images identical to routine MDCT images were generated and reformatted in a sagittal orientation and a section thickness of 3 mm, based on a standard bone filter (YB). CT imaging data post-processing took around 3 ± 1 min for each examination, consisting of around 2 ± 1 min for SBI reconstruction routinely performed by the CT workstation, 20 s in total for data transfer, 10 s for calculating monoenergetic images and another 30 s for the creation of material-specific density maps.

### CT imaging analysis and fracture age determination

Conventional CT images were independently assessed by two radiologists (A.S.G. and B.J.S., both with 6 years of experience) blinded to spectral CT imaging information, MR imaging findings, and clinical information. Vertebral fracture age was assessed according to standard fracture signs used with CT [23]. The radiologists were allowed to scroll through sagittal reformations and to adjust the window and level settings for personal convenience. A four-point Likert scale was used to describe the individual fracture age assessment (1 = chronic fracture; 2 = likely chronic fracture; 3 = likely acute fracture; 4 = acute fracture). For contingency tables, categories 1 and 2 were summarized as non-acute fracture and 3 and 4 as acute fracture. In subjects with multiple vertebral fractures, the fracture age was assessed separately for each vertebra.

After at least 4 weeks, the same readers assessed material-specific density maps derived from dual-layer spectral CT images in a random order, again blinded for all other information. Radiologists used all available density maps (red marrow, yellow marrow, and hydroxyapatite volume fraction), and again were allowed to freely scroll through sagittal reformations and to adjust the window and level settings for personal convenience. Regions with increased red marrow and reduced yellow marrow density, indicating bone-marrow edema equivalent changes were assessed by using a Likert scale similar to the one applied to conventional CT images (1 = no edema; 2 = likely no edema; 3 = likely edema; 4 = edema). Again, for contingency tables, categories 1 and 2 were summarized as non-acute fracture and 3 and 4 as acute fracture.

### MR imaging as standard of reference

All subjects were examined at our institution using a 3-T MR system (Verio, Siemens Healthineers, Erlangen, Germany) within 3 days after admission. Protocols comprised sagittal short tau inversion recovery (TR, 4000 ms; TE, 270 ms; TI, 160 ms; echo train length, 16; flip angle 180°; slice thickness, 3 mm; matrix, 448 × 448), T2-weighted turbo spin echo (TSE; TR, 3000 ms; TE, 113 ms; echo train length, 21; flip angle 180°; slice thickness, 3 mm; matrix, 384 × 384) and T1-weighted TSE (TR, 693 ms; TE, 13 ms; echo train length, 3; flip angle 180°; slice thickness, 3 mm; matrix, 384 × 384) sequences as well as an axial T2-weighted TSE sequence (TR, 5240 ms; TE, 113 ms; echo train length, 19; flip angle 176°; slice thickness, 3 mm; matrix, 320 × 320). According to standard-of-care criteria [23], MR images were assessed for the presence of traumatic bone marrow edema by a third reader (T.B., board-certified radiologist with 8 years of experience in spine imaging), again blinded for all other imaging and clinical information. A binary classification was used (0 = normal bone marrow; 1 = bone marrow edema equivalent signal changes) for this standard of reference.

### Statistical analysis

Statistical analyses were performed with SPSS 24 (IBM; Armonk, NY, USA). A *P* value of less than 0.05 was considered to indicate a statistically significant difference.

In addition to descriptive statistics, independent-samples* t* tests (for continuous variables) and Chi-squared tests (for dichotomous variables) were used to compare patient characteristics. Sensitivity, specificity, accuracy, the positive and negative predictive values of the assessment of acute vertebral fractures in conventional and dual-layer spectral CT images were assessed in contingency tables, both on the level of individual fractures (Table 1) and of patients (Table 2). For the latter, a patient with at least one acute fracture as identified on MRI was categorized as “acute”, and the detection of at least one acute fracture on conventional CT or DLCT, respectively, was categorized as “acute”. The interreader agreement for Likert scales was assessed with weighted Cohen’s κ statistics.

**Table 1:** Contingency table for the classification of vertebral fractures as acute vs. non-acute based on conventional CT images, dual-layer spectral CT images, and MR imaging as the standard of reference, displayed for two radiologists

	Radiologist 1			
	Considered acute on conventional CT		Considered acute on dual-layer spectral CT	
Edema-like signal on MR imaging	Acute	Non-acute	Acute	Non-acute
Edema-like signal (acute;* n* = 41)	31	10	38	3
No edema-like signal (non-acute;* n* = 18)	4	14	2	16
	Radiologist 2			
	Considered acute on conventional CT		Considered acute on dual-layer spectral CT	
Edema-like signal on MR imaging	Acute	Non-acute	Acute	Non-acute
Edema-like signal (acute;* n* = 41)	30	11	39	2
No edema-like signal (non-acute;* n* = 18)	3	15	2	16

**Table 2:** Diagnostic performance of conventional and dual-layer spectral CT for the detection of acute vertebral fractures with MR imaging as standard-of-reference on the level of individual fractures (*n* = 59)

	Radiologist 1		Radiologist 2	
Detection of acute fractures	Conventional CT	Dual-layer spectral CT	Conventional CT	Dual-layer spectral CT
Sensitivity	0.76	0.93	0.73	0.95
Specificity	0.78	0.89	0.83	0.89
Accuracy	0.76	0.92	0.76	0.93
Positive predictive value	0.89	0.93	0.91	0.95
Negative predictive value	0.58	0.84	0.58	0.89

## Results

### Subjects and fracture status

In 27 patients (mean age, 72 ± 11 years; 17 women), a total of 59 vertebral fractures were identified. According to MR findings as standard of reference, 41 fractures (70%) were categorized as acute fractures. Of 27 patients, 23 had at least one fracture as identified on MRI. Twenty-nine fractures (49%) were located in the thoracic spine, and 30 fractures (51%) were located in the lumbar spine, with the highest prevalence in Th12 (17%) and L1 (15%). No significant differences were found regarding age and sex between patients with and without at least one acute fracture (*P* > 0.05 for both).

### Performance of conventional and dual-layer spectral CT

Of 41 fractures that were considered to be acute, 31 were correctly identified as such on conventional CT images by radiologist 1 (radiologist 2, *n* = 30), and *n* = 38 were correctly identified as such on dual-layer spectral CT images by radiologist 1 (radiologist 2, *n* = 39) (Table 1). Analogously, conventional CT images showed a sensitivity of 0.73–0.76 and specificity of 0.78–0.83, whereas the sensitivity (0.93–0.95) and specificity (0.89) based on dual-layer spectral CT images were substantially higher. Accuracy increased from 0.76 for conventional CT images to 0.92–0.93 using dual-layer spectral CT images (Table 2).

**Fig. 2:**
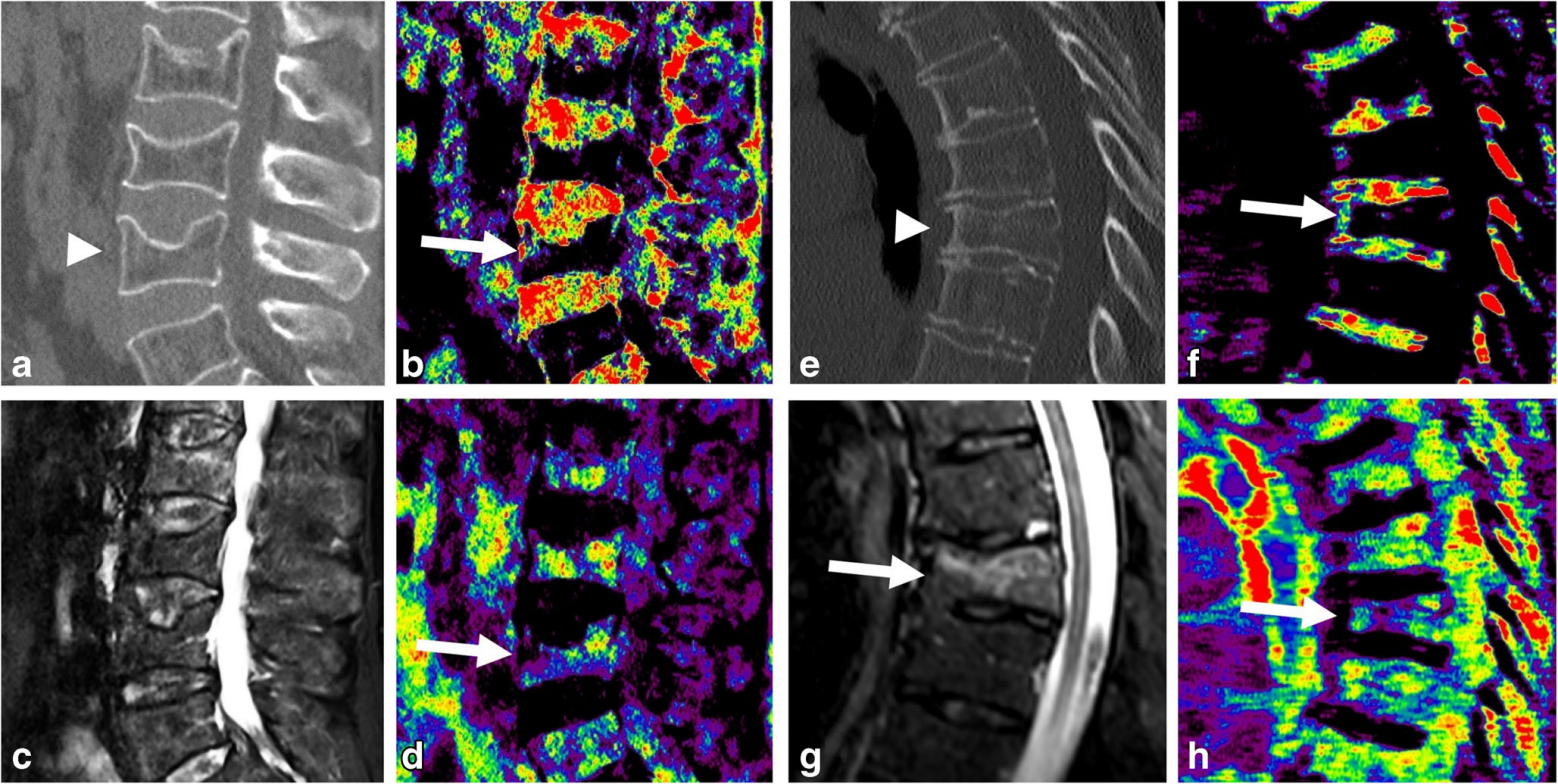
CT and MR images of exemplary false-positive (**a**–**d**) and false-negative (**e**–**h**) classifications based on dual-layer spectral CT imaging. The L4 endplate fracture in a 73-year-old male patient with acute back pain (**a**) was considered acute based on a water-specific density increase ventrally in the vertebral body (**b**;* arrow*) and fat-specific density decrease (**d**;* arrow*); however, the fat-saturated fluid-sensitive STIR sequence (**c**) reveals no edema-equivalent signal alteration. The Th8 compression fracture in a 72-year-old female patient with acute back pain and known osteopenia (**e**) was considered chronic due to absent definite water- and fat-specific density changes in comparison to the adjacent vertebral bodies (**f** and** h**;* arrows*); however, the STIR sequence revealed an extensive edema-equivalent signal alteration indicating an acute fracture

On a patient level, in individuals with at least one acute vertebral fracture as identified on MRI at least one fracture was classified as acute based on conventional CT images in 19 of 23 cases, while this number increased to 23 based on dual-layer spectral CT (Table 3). Subsequently, on a patient level, the sensitivity and the negative predictive value of CT-based fracture assessment both increased to 1, respectively, using dual-layer spectral CT data. Examples of false-positive and false-negative cases are shown in Fig. 2.

**Table 3:** Diagnostic performance of conventional and dual-layer spectral CT for the detection of at least one acute vertebral fractures with MR imaging as standard-of-reference on a patient level (*n* = 27; parameters are identical for radiologist 1 and 2)

Detection of acute fractures	Conventional CT	Dual-layer spectral CT
Sensitivity	0.83	1
Specificity	0.75	0.75
Accuracy	0.81	0.96
Positive predictive value	0.95	0.96
Negative predictive value	0.43	1

Interreader agreement for the assessment of fractures with four-point Likert scales was excellent for both conventional CT images (weighted κ = 0.81; 95% asymptotic confidence interval (CI), 0.70, 0.92) and dual-layer spectral CT images (weighted κ = 0.96; 95% CI, 0.92, 1.00).

## Discussion

In our study, the detection of bone marrow edema, and thus the identification of patients with acute thoracolumbar vertebral fractures, was feasible using three-material decomposition and material-specific density maps calculated from dual-layer spectral CT. The accuracy, sensitivity, and specificity for the diagnosis of acute vertebral fractures was substantially higher with three-material decomposition generated from dual-layer spectral CT images compared to the conventional CT images, using MR imaging as standard of reference.

Depending on the hardware setup, there are different ways to generate spectral information based on dual-energy CT. Dual-energy with dual-source CT, using either different characteristics of two different X-ray sources or fast kV switching between two different kV settings, has been commercially available for years now [8–10]. More recently, dual-layer spectral CT as used in this study has been implemented in clinical routine, using two different detector layers stacked upon each other of which each layer absorbs a different energy spectrum [11]. The dual-layer spectral CT technique enables material decomposition and material-specific density measurements by exploiting the material and energy-dependent X-ray absorption [10]. Unlike with dual-energy CT, spectral CT allows the collection and analysis of spectral information without the use of a predefined specific protocol, whereas dual-energy CT requires a preselection of specific settings for the anatomic region and pathology of interest. Previously, the three-material decomposition model has been used to optimize the diagnostic performance of dual-source, dual-energy CT by calculating virtual non-calcium images, and several studies have reported on the virtual non-calcium imaging technique for the visualization of bone marrow edema in the spine so far [16–18, 24]. Most of these previous studies analyzed images which were obtained with first- or second-generation dual-source, dual-energy CT scanners, while a recent study by Petritsch et al. assessed the diagnostic performance of virtual non-calcium maps based on data from third-generation dual-energy CT [15]. The accuracy for the detection of acute fractures as reported in their study was 0.94, which is comparable to our findings. In another recent study by Kaup et al., in which different second- and third-generation dual-source, dual-energy CT scanners operating in different scanning modes were assessed, the diagnostic accuracy was lower than in the study by Petritsch et al. and our analysis [16]. This suggests that bone marrow edema visualization may depend on several technical factors and also the personal routine of the radiologist regarding the assessment of material-specific density maps.

A previous study using spectral CT imaging information, obtained by using a dual-layer spectral CT scanner, demonstrated that calcium-hydroxyapatite (HA) imaging was feasible in human vertebrae [25]. Moreover, different degrees of obesity showed no significant effect on the quantification of calcium-hydroxyapatite (HA) concentrations as well as HA-specific BMD measurements in vertebral specimens, using conventional qCT measurements as well as reference phantoms as the standard of reference [25]. Analogous to this previous publication, we used standard clinical examination protocols without specific presets.

In so doing, we found the diagnostic performance for the detection of bone marrow edema of this novel approach to be comparable to the performance of third-generation dual-source, dual-energy CT scanners as reported recently [15].

Given the increased accuracy of fracture assessment based on dual-layer spectral CT, radiologists may be more confident regarding the classification of a vertebral fracture as acute or chronic without MRI. Since MR is associated with additional examination times and costs and may not be available within a timely manner, this could be beneficial to the majority of patients in an acute clinical setting as well as for patients with MR contraindications.

Post-processing of CT imaging data were performed on a software suite commercially available from the CT manufacturer. Of note, this was done for convenient imaging data handling. However, the post-processing algorithm as described in the Methods section may be implemented in any other freely or commercially available software using any other programming language that allows image handling and algebraic operations; therefore, the presented method for material decomposition and creation of material-specific density maps may be applied to any dual-energy CT imaging data and in any software environment.

Our study population age range was fairly wide and comparable to the range reported in previous studies [18, 24]. In older patients that present with osteoporotic fractures, typically the fat content of bone marrow is substantially higher than in younger subjects [24, 26], thus facilitating the differentiation of bone marrow edema from bone marrow with a high fat content. In younger patients with a higher percentage of red bone marrow, the depiction of bone marrow edema may be more difficult. In future studies, the effects of bone marrow composition in different age groups on the performance of bone marrow edema detection based on dual-layer spectral CT images needs to be assessed in order to evaluate the age-dependency of this approach for bone marrow edema visualization.

This study has further limitations. Since we focused on traumatic fractures in an older patient collective, we excluded patients with other conditions associated with bone marrow changes such as metabolic diseases, other than osteoporosis, or malignant entities. The evaluation of bone marrow changes in these populations should be assessed in future studies, also with a particular focus on the differentiation of benign and malignant fractures. Moreover, the study population of this retrospective analysis was relatively small due to the novelty of the dual-layer spectral CT system and the fact that only subjects undergoing both dual-layer spectral CT and MR imaging within 3 days were included. In the same context, only patients with a clinically suspected acute fracture were admitted to CT imaging and thus could be included, therefore, in our study population, substantially more acute fractures were found than non-acute fractures and 23 of 27 patients had at least one fracture identified as acute on MRI. Therefore, the significance of analyses on a patient level may be limited. In contrast, comparing the performance of conventional CT and dual-layer CT on the level of individual vertebral bodies not only increased the number of samples but also may be more appropriate in a clinical setting in which the majority of osteoporotic patients present not only one either acute or old vertebral fracture, but rather several either acute or old vertebral fractures, and the classification of individual fractures may be relevant for therapy decisions. Finally, only one dual-energy system, i.e., dual-layer spectral CT, was assessed in this study, and only one material decomposition algorithm was used. Therefore, the performance of the presented method can be compared to the numbers available from previous publications such as Petrisch et al. and Kaup et al. [15, 16]; however, the direct comparison of different setups has not been a part of this analysis and should be investigated further in the future.

In conclusion, our study showed that the detection of bone marrow edema and thus acute vertebral fractures based on three-material decomposition generated from dual-layer spectral CT images was feasible with a substantially higher accuracy compared to conventional CT images. This suggests that the presented method may spare patients additional examinations and facilitate the diagnosis of vertebral fractures.
